# Depressive Symptoms during an Acute Schizophrenic Episode: Frequency and Clinical Correlates

**DOI:** 10.1155/2015/674641

**Published:** 2015-11-18

**Authors:** Ravi Philip Rajkumar

**Affiliations:** Department of Psychiatry, Jawaharlal Institute of Postgraduate Medical Education and Research (JIPMER), Pondicherry 605 006, India

## Abstract

*Introduction*. Depressive symptoms are common in schizophrenia and are associated with poorer functioning, lower quality of life, and an elevated risk of suicidal behaviour. There are few studies on the occurrence and correlates of these symptoms in acutely ill patients with schizophrenia.* Method.* 72 acutely ill patients with schizophrenia were assessed for depression using the Calgary Depression Scale for Schizophrenia (CDSS). A cut-off score of ≥6 on the CDSS was used to identify clinically significant depressive symptoms. The relationship between depression and illness variables, including psychotic symptom dimensions as measured by the Positive and Negative Syndrome Scale for Schizophrenia (PANSS), was examined.* Results*. Eleven (15.3%) patients had clinically significant depressive symptoms. These patients scored higher on the positive and general psychopathology scales of the PANSS and had higher rates of suicidal behavior and poorer functioning. The severity of depressive symptoms was positively correlated with the PANSS positive subscale and negatively correlated with the PANSS negative subscale.* Discussion*. These findings confirm previous reports that depressive symptoms in active schizophrenia is related to the severity of positive psychotic symptoms and is a risk factor for suicidal behaviour in these patients.

## 1. Introduction

Depressive symptoms are often seen in patients with schizophrenia. These symptoms may occur during the prepsychotic or “prodromal” phase [1, 2], during initial episodes of schizophrenia [3], in chronically ill patients [4], and even in patients who have been stabilized on treatment [5, 6]. In a substantial number of patients, these symptoms are severe enough to qualify for a syndromal diagnosis of depression as per standard diagnostic criteria [1, 5].

Some researchers have suggested that depressive symptoms may be a distinct symptom dimension in the complex syndrome of schizophrenia [7]. They may also be related to distress over symptoms such as paranoid delusions [8] or experiences of stigma that have been internalized by the patient [9]. Variables typically associated with depressive disorder, such as gender, appear to have little influence on the development of depressive symptoms in schizophrenia [10].

Regardless of the mechanisms underlying their development, depressive symptoms in schizophrenia are of considerable clinical importance. They are associated with a higher risk of suicide attempts [11, 12], lower quality of life [4, 13, 14], poorer functional outcomes [15, 16], and possibly a longer amount of time spent hospitalized [17]. Moreover, they are often undetected for a variety of reasons [18].

Given the above implications, there is a need to better understand the clinical variables associated with depressive symptoms in the various phases of schizophrenia. Existing research suggests that during an acute episode of schizophrenia, depression is associated with positive symptoms of psychosis [19], while in a chronic or stable phase it may be better correlated with negative symptoms [6, 19]. Relatively few studies have been carried out in patients during an acute episode. The current study aims to add to this finding by assessing the clinical correlates of depressive symptoms in a hospital-based sample of patients presenting with an acute episode of schizophrenia.

## 2. Methods

Consecutive in- or out-patients presenting to the psychiatric services of our institute with an acute episode of schizophrenia were recruited over the period from January 2013 to December 2014. Inclusion criteria were (a) a diagnosis of schizophrenia as per the Diagnostic and Statistical Manual, Fourth Edition, Text Revision (DSM-IV-TR) guidelines [20], as confirmed by a consultant psychiatrist following evaluation by a resident doctor, (b) an acute episode, defined as any new-onset or recurrent psychotic symptoms which required either initiation of antipsychotic treatment, change in existing treatment, or hospitalization, (c) age of 18 to 60 years, and (d) written informed consent from the patient or his/her primary caregiver. We did not include patients with schizoaffective disorder (*n* = 3 during the study period), as the affective symptoms exhibited by these patients would have confounded the interpretation of our results. A total of seventy-two patients were included in this study.

Basic demographic information (age, gender, and marital and educational status) and details of the course of the patient's illness, such as age of onset and number of episodes, were obtained by interviewing patients and caregivers, supplemented by their existing medical records. All patients' psychotic symptoms were rated using the Positive and Negative Syndrome Scale for Schizophrenia (PANSS) [21], and their current level of overall functioning was estimated with the Global Assessment of Functioning Scale [22].

Depressive symptoms were rated using the Calgary Depression Scale for Schizophrenia (CDSS). This clinician-rated instrument was specifically designed to measure depression in patients with schizophrenia, while avoiding overlap with negative symptoms or drug-induced extrapyramidal side effects [23]. It has been found to be more specific than standard depression rating scales, such as the Hamilton Rating Scale for Depression, in this population [24]. A systematic review of instruments to assess depression in schizophrenia found that the CDSS was the best existing option for both clinical and research purposes [25].

A CDSS score of 6 or more has generally been used as a cut-off value to indicate clinically significant depressive symptoms [16, 26], and we used this value, in addition to the total CDSS score, for data analysis in our study. Patients with scores above and below this cut-off were compared on categorical variables (such as gender or diagnostic subtype), using the* chi-*square test or Fisher's exact test with Bonferroni's correction where multiple comparisons were made, and on continuous variables (such as PANSS and GAF scores), using the independent samples *t*-test or the Mann-Whitney *U* test. Correlations between depression scores and other measures of psychopathology were assessed using Spearman's correlation coefficient. All tests were two-tailed, and a value of *p* < 0.05 was considered significant. As this study was exploratory in nature, there was no* a priori* hypothesis.

## 3. Results

The final study sample consisted of 72 patients, 42 women and 30 men. The majority of the samples (*n* = 60, 83.3%) were hospitalized at the time of assessment. The mean age of the sample at presentation was 32.68 ± 8.24 years (range 18 to 53 years), and the majority of patients (*n* = 53, 73.6%) were in their first episode of schizophrenia at the time of evaluation.

The mean CDSS score of these patients was 2.76; however, these scores were not normally distributed, and a substantial minority (*n* = 11 patients, 15.3%) had a CDSS score of 6 or greater, indicating clinically relevant depressive symptoms. Comparisons between these patients and the rest of the study sample are summarized in Table 1. Patients with significant depressive symptoms had higher rates of both total and violent lifetime suicide attempts (*p* < 0.01 in both cases, Fisher's exact test) and made more lifetime suicide attempts (Mann-Whitney *U* = 599.5, *p* < 0.01). They had significantly higher scores on the PANSS positive and general psychopathology subscores, but not the total PANSS score. The PANSS psychopathology index, calculated using the formula (PANSS positive score – PANSS negative score), was also significantly higher in these patients.

Though both groups had low levels of functioning due to their active psychotic symptoms, the group with significant depressive symptoms had poorer functioning (Mann-Whitney *U* = 175.0, *p* = 0.012). Comorbid obsessive-compulsive disorder was found in two patients in the depressed group, but in none of the remainder; this difference was not statistically significant after applying Bonferroni's correction for multiple comparisons.

In addition to the analysis in Table 1, we also examined the correlations between the total CDSS score and measures of psychopathology and functioning. The CDSS score was positively correlated with the PANSS positive (Spearman's *ρ* = 0.335, *p* < 0.01) and general psychopathology (Spearman's *ρ* = 0.272, *p* = 0.021) subscores; on the other hand, it was significantly negatively correlated with the PANSS negative syndrome subscore (Spearman's *ρ* = −0.365, *p* < 0.01). PANSS general item G6 (depression) was highly positively correlated with the CDSS score, indicating good convergence between the two measures of depression (Spearman's *ρ* = 0.522, *p* < 0.01). Finally, the GAF total score and the CDSS total score were inversely correlated with each other (Spearman's *ρ* = −0.317, *p* < 0.01).

## 4. Discussion

Patients in our sample had generally low levels of depressive symptoms, comparable to those obtained when the CDSS was applied to a general population sample [27]. However, over 15% of them had clinically relevant symptoms of depression. These findings are consistent with earlier published literature, which indicates that the majority of patients with schizophrenia do not have depression at a syndromal level [3, 5, 6, 28]. Our prevalence of 15% in patients with an acute schizophrenic episode is comparable to the rate found in a sample of Chinese patients with active first-episode psychosis (15.1%) [26] but is much lower than reported rates in other studies of patients with active psychosis [3, 19]. The reasons for this discrepancy are unclear but may be related to study settings, inclusion criteria, and differing criteria used to define significant depressive symptoms.

Patients with significant levels of depression had significantly higher levels of suicidal behaviour, whether this was defined categorically or in terms of the number of suicide attempts. The association between depressive symptoms and suicidality in schizophrenia is well-documented in the literature [11, 12, 29–31] and our findings are in keeping with them.

Depressive symptoms had a specific relationship to the dimensions of psychosis in our patient sample. Regardless of whether these symptoms were defined continuously or using a cut-off, they were positively associated with positive symptoms. This relationship has been found in prior studies of patients with active psychosis [8, 19, 26]. It has been suggested that specific mood-based symptoms, such as guilt, lead to the development of paranoid delusions [32]. However, we could not establish the causal direction of the relationship between depression and delusions in this study due to its cross-sectional nature. Alternately, depressive symptoms and delusions may share some, but not all, causal mechanisms at the level of information processing [33], which may explain the positive correlation between them.

Conversely, depression was inversely related to measures of negative symptoms. This is an unexpected finding, as negative symptoms have been found to be positively correlated with depression in stable patients with chronic schizophrenia [6]. A possible explanation for this is that negative symptoms may represent an avoidant defence mechanism against the psychological distress and trauma caused by the experience of positive psychotic symptoms [34] and that depressive symptoms may arise when such a defence fails.

Depressive symptoms were associated with poor cross-sectional functioning, a finding which remained significant even though mean GAF scores were low across the entire sample. This is consistent with earlier literature indicating that depression is associated with poorer functional outcomes [15, 16, 35] and highlights the need for an improved understanding and more effective management of this syndrome in patients with schizophrenia.

Our results are subject to certain limitations. First, we did not assess predisposing variables of interest, such as childhood trauma, which are related to depressive symptoms in psychosis [36]. Second, we did not examine the potential influence of treatment, such as typical or atypical antipsychotics, in either worsening or ameliorating depression [37, 38]. Third, we did not conduct an in-depth assessment of insight, which is an important correlate of depression in schizophrenia. Fourth, we did not assess patients' subjective or objective quality of life, which are also known to be affected by depression. Fifth, owing to the cross-sectional nature of our study, we could not assess the relationship between depressive symptoms and other psychotic symptom dimensions over time. Finally, owing to the small sample size, the conclusions that can be drawn from our data are limited.

In conclusion, our study underlines the fact that depressive symptoms are a significant clinical problem in patients presenting with an acute psychotic episode and have specific relationships to other domains of psychopathology. The possible psychological mechanisms underlying these associations still remain speculative but merit further exploration. This would lead to improvement in the management of depressive symptoms, leading to improved functioning and a reduced risk of suicide.

## Figures and Tables

**Table 1:** Comparisons between patients with and without clinically significant depressive symptoms.

Variable	Patients with significant depressive symptoms (*n* = 11)	Patients without significant depressive symptom (*n* = 61)
Age at presentation, years	32.9 (8.41)	32.63 (8.28)
Gender		
Male	5 (45%)	25 (41%)
Female	6 (55%)	36 (59%)
Marital status		
Single	4 (36%)	29 (48%)
Married	6 (55%)	30 (49%)
Divorced or separated	1 (9%)	2 (3%)
Years of formal education	10.45 (3.86)	9.67 (3.44)
Place of residence		
Urban	7 (64%)	31 (51%)
Rural	4 (36%)	30 (49%)
Age at onset, years	27.45 (4.5)	27.03 (8.18)
Duration of illness, years	2 (0.5–22)	4 (0.5–30)^†^
Number of hospitalizations	1.55 (1.51)	1.16 (0.69)
Total duration of hospitalization, days	36.6 (33.67)	25.61 (19.8)^¶^
First-episode schizophrenia	9 (82%)	44 (72%)
Subtype of schizophrenia		
Paranoid	9 (82%)	27 (44%)
Catatonic	—	6 (10%)
Hebephrenic	—	2 (3%)
Undifferentiated	2 (18%)	26 (43%)
Presence of specific psychotic symptoms		
Delusions, any	11 (100%)	44 (72%)
Hallucinations, any	8 (73%)	48 (79%)
Catatonic symptoms	—	17 (28%)
Disorganized speech or thought	1 (9%)	5 (8%)
Disorganized behaviour	2 (18%)	23 (38%)
PANSS scores		
Positive subscale	23.27 (3.16)	19.38 (4.66)^*∗∗*^
Negative subscale	15.81 (6.71)	19.87 (7.84)
General psychopathology subscale	47.18 (9.25)	39.9 (7.82)^*∗∗*^
Psychopathology index	7.45 (6.76)	−0.49 (10.0)^*∗∗*^
Total	86.27 (14.01)	79.14 (14.73)
Suicide attempt, lifetime	9 (82%)	11 (18%)^*∗∗*^
Violent suicide attempt, lifetime	7 (64%)	7 (12%)^*∗∗*^
Number of suicide attempts	1.91 (1.64)	0.33 (0.87)^*∗∗*^
GAF score	23.55 (6.25)	29.28 (6.36)^*∗*^
Body mass index, kg/m^2^	23.97 (4.35)	22.92 (4.24)
Comorbid diagnoses		
Obsessive-compulsive disorder	2 (18%)	—
Nicotine dependence	3 (27%)	11 (18%)
Alcohol dependence	—	2 (3%)

All values given as mean (standard deviation) or frequency (percentage).

PANSS: Positive and Negative Syndrome Scale for Schizophrenia; GAF: Global Assessment of Functioning Scale.

^*∗*^Significant at *p* < 0.05.

^*∗∗*^Significant at *p* < 0.01.

^¶^Calculated for a total of 64 patients who had been hospitalized, 54 without and 10 with significant depression.

^†^Given as median (range).
